# Red Man Syndrome Following Intraperitoneal Vancomycin in a Child with Peritonitis

**DOI:** 10.3389/fped.2014.00055

**Published:** 2014-06-05

**Authors:** Melissa J. Domis, Michael L. Moritz

**Affiliations:** ^1^Department of Pediatrics, Cleveland Clinic Children’s, Cleveland, OH, USA; ^2^Division of Nephrology, Department of Pediatrics, Children’s Hospital of Pittsburgh of UPMC, University of Pittsburgh School of Medicine, Pittsburgh, PA, USA

**Keywords:** peritonitis, vancomycin, red man syndrome, peritoneal dialysis, adverse reaction

## Abstract

Red man syndrome (RMS) has frequently been reported to occur with intravenous vancomycin therapy. However, there have been few reports of this complication during intraperitoneal (IP) treatment with vancomycin. This report describes an 11-year-old boy with end stage renal disease who developed RMS 45 min into the initial loading dose of IP vancomycin for the treatment of bacterial peritonitis with a vancomycin level of 38.8 mcg/mL. The patient developed this adverse reaction despite appropriate initial loading dose per ISPD guidelines for continuous treatment (1000 mg/L). This case emphasizes the importance of monitoring for adverse reactions of vancomycin therapy, and raises dosing considerations that differ slightly from the currently recommended ISPD guidelines for IP vancomycin treatment in the treatment of bacterial peritonitis.

## Introduction

Bacterial peritonitis is one of the most frequent infectious complications of peritoneal dialysis with significant morbidity. The current ISPD guidelines for treatment of peritonitis in pediatric patients recommend intraperitoneal (IP) glycopeptides (either vancomycin or teicoplanin, which is unavailable in the United States) for Gram-positive coverage and IP third or fourth generation cephalosporin for Gram-negative coverage (1). The recommended initial IP vancomycin dose is a 1000 mg/L loading dose followed by 25 mg/L for maintenance therapy or 30 mg/kg as a loading dose followed by 15 mg/kg every 3–5 days for intermittent therapy (1). This loading dose of vancomycin is far higher than intravenous doses recommended to treat other bacterial infections, 10–15 mg/kg. Potential adverse reactions to vancomycin include ototoxicity, nephrotoxicity, neutropenia, and phlebitis (2). One potential adverse reaction of using such a large initial dose of vancomycin, given all at once, is red man syndrome (RMS). RMS is the most common toxicity of intravenous vancomycin therapy and is associated with rapid infusion of large doses of vancomycin (2, 3). The reaction occurs secondary to histamine release from mast cells and can manifest as generalized flushing, pruritus, erythematous rash, chest pain, dyspnea, and hypotension (2, 3). Cardiac toxicity has been reported to be severe enough to cause cardiac arrest (2). We present the case of an 11-year-old boy who developed RMS shortly following the initial loading dose of vancomycin for the treatment of bacterial peritonitis. We also review the literature that ISPD dosing recommendations for IP vancomycin are based on and suggest the need for further investigation of dosing in children, as the currently recommended dose may be too large for some patients.

## Case Report

An 11-year-old 37.3 kg Amish boy with end-stage renal disease secondary to posterior urethral valves managed with continuous ambulatory peritoneal dialysis (CAPD) for 2 years presented to the hospital for the evaluation of peritonitis due to cloudy peritoneal fluid. His CAPD prescription consisted of four exchanges with a fill volume of 1400 mL of 1.5% Dianeal. Eight months prior, he had received 750 mg (20 mg/kg) of IP vancomycin antibiotic prophylaxis for a laceration of his PD catheter. Cultures subsequently grew coagulase-negative *Staphylococcus* and *Enterococcus faecalis*, without clinical evidence of peritonitis, which was successfully treated with 500 mg (13 mg/kg) of intermittent IP vancomycin. He had no reaction to the IP vancomycin, and he had no previous medication allergies. On presentation he was well-appearing, afebrile, in no acute distress, and without abdominal pain or tenderness. The PD catheter exit site and tunnel were intact. The peritoneal cell count and differential revealed white blood cell count of 2165/mm^3^, red blood cell count of 5/mm^3^ with a differential of 75% neutrophils, 17% lymphocytes, 6% monocytes, and 2% eosinophils. Peritoneal fluid cell count and differential were consistent with peritonitis.

Empiric treatment with IP vancomycin and cefepime were initiated as per ISPD guidelines (1). Vancomycin was administered IP at a concentration of 1000 mg/L in a 1400 mL long dwell (using the standard 40 mL/kg dwell volume for a child this age), equivalent to a total dose of 37.5 mg/kg. Approximately 45 min into the dwell, the patient developed rash, itching, and agitation consistent with RMS. The patient was hemodynamically stable. The PD fluid was drained and the patient was treated with oral Benadryl (25 mg). The vancomycin level at this time was 38.8 mcg/mL. The patient clinically improved with Benadryl.

Ultimately, peritoneal dialysis fluid cultures grew coagulase-negative *Staphylococcus* and cefepime treatment was discontinued. The patient was subsequently treated in-house with 30 mg/L vancomycin to PD and was sent home on day 3 of admission with intermittent IP vancomycin 350 mg/L or total dose of 15 mg/kg/dose (500 mg) every 3 days for 14 days. Random vancomycin trough levels were drawn prior to IP vancomycin administration for close monitoring. Patient had no further episodes of RMS during vancomycin therapy.

## Discussion

In this report, we described a child who developed RMS soon after the initiation of IP vancomycin as per ISPD guidelines (1). After a dwell time of only 45 min, the vancomycin level was elevated at 38.8 mcg/mL. Despite receiving a dose in accordance with the ISPD guidelines for continuous treatment (1000 mg/L), the total dose administered (37.5 mg/kg) was in excess of the recommended loading dose of the ISPD for intermittent therapy (30 mg/kg) (1). He had not developed RMS when he was treated with IP vancomycin at a dose between 15 and 20 mg/kg. Upon review of the literature, there was only one previous report of RMS as a side effect of IP vancomycin therapy (4). This article, which was reviewed as part of the ISPD Consensus Guidelines, noted that the large loading dose of IP vancomycin necessary to obtain therapeutic serum levels is not without risk of side effects. The ISPD guidelines do not mention RMS as a potential complication (1).

Schaefer et al. performed a controlled, prospective study of IP antibiotics in treatment of active peritonitis in 152 children and reported three cases of RMS during the IP vancomycin loading dose dwell with vancomycin levels as high as 72 mcg/mL (4). The continuously treated group in this article was treated with a loading dose of only 15 mg/kg IP, a dose well below the 37.5 mg/kg that our patient received, and adverse effects and elevated vancomycin levels were still reported (4). This raises the question if the high loading doses of 1000 mg/L and 30 mg/kg IP is in excess of what is necessary to achieve an adequate therapeutic vancomycin level, and if the guidelines should be revised to ensure that the continuous therapy loading dose does not exceed 30 mg/kg? Consideration should be given to limiting the initial loading dose to 15 mg/kg and administering an additional 15 mg/kg if there is a subtherapeutic vancomycin level. The dosing of vancomycin that the ISPD recommends was determined from extrapolation models from adult pharmacokinetic studies in patients without active peritonitis that excluded patients with a history of vancomycin sensitivity (1, 5). The efficacy of the recommended loading dose has since been demonstrated in various studies with children but both caution against the potentially high serum levels of vancomycin (4, 6). Furthermore, Blowey demonstrated marked variability in peritoneal transport of vancomycin, suggesting that transport status and thus rapidity of vancomycin uptake varies from patient to patient (6). Because treatment is continuous and vancomycin transport times vary, vancomycin blood levels cannot reflect a peak or trough level of the drug with certainty. Thus, the recommended doses of the ISPD may not be appropriate for all patients.

Although RMS usually occurs with the first dose of vancomycin, it can occur at any time even in a patient with previous exposure to the medication (3). This patient had previous tolerated IP vancomycin well, but at a much lower dose. This is particularly an issue for patients undergoing PD receiving intermittent therapy with IP vancomycin 30 mg/kg every 5–7 days as an outpatient. As the goal of the ISPD guidelines is to recommend the therapy with the highest efficacy and lowest potential for complications, consideration should be given to specifically mentioning RMS as a side effect in the next revision of the ISPD guidelines (1). This would help to guide the physician in advising families what adverse reactions to watch for at home. It may also be relevant to further review the literature and include recommendations for prophylaxis to avoid the complication of RMS, both in the hospital and as an outpatient, given the risk for hypotension and potentially cardiac arrest. Wazny and Daghigh suggest prophylaxis with H1 receptor antagonist (IV diphenhydramine 1 mg/kg, oral hydroxyzine 50 mg, oral diphenhydramine 50 mg) to decrease histamine release and help prevent RMS (3).

Although not available in the United States, teicoplanin is a safer and equally effective alternative to vancomycin for IP treatment of Gram-positive organisms in peritonitis. The Schaefer et al. study mentioned above also compared groups treated with vancomycin and teicoplanin and documented no hypersensitivity reactions in the group treated with IP teicoplanin (4). Schaefer’s study also suggests that teicoplanin, an equally effective glycopeptides, has a safer overall side effect profile than IP vancomycin. Various other studies have reported the safety and efficacy of teicoplanin compared to vancomycin (7–9). Thus, teicoplanin may be considered for use in the future as a safer IP antibiotic to treat Gram-positive peritonitis or as an option for children with past hypersensitivity reactions to vancomycin if the drug becomes more widely available in the United States.

In summary, this report describes a case of RMS developing following the appropriate initial dose of IP vancomycin for the treatment of peritonitis in a child. This case demonstrates the need to closely monitor for side effects of IP vancomycin therapy and to carefully consider the dosing of IP vancomycin in patients being treated for bacterial peritonitis. When following the ISPD guidelines for loading dose of vancomycin for continuous treatment, it is possible to exceed the loading dose recommended for intermittent therapy if the child’s dwell volume is large because of patient’s weight. A brief review of the literature also highlights that vancomycin levels in various studies exceeded those of the patient described here, even with lower loading doses of vancomycin. Consideration should be given to lowering the loading dose of IP vancomycin to avoid side effects and supratherapeutic serum vancomycin levels.

## Conflict of Interest Statement

The authors declare that the research was conducted in the absence of any commercial or financial relationships that could be construed as a potential conflict of interest.
